# Age-Dependent Myocardial Dysfunction in Critically Ill Patients: Role of Mitochondrial Dysfunction

**DOI:** 10.3390/ijms20143523

**Published:** 2019-07-18

**Authors:** Andrew J. Lautz, Basilia Zingarelli

**Affiliations:** Division of Critical Care Medicine, Cincinnati Children’s Hospital Medical Center, and Department of Pediatrics, College of Medicine, University of Cincinnati, Cincinnati, OH 45229, USA

**Keywords:** sepsis, cardiac arrest, age, echocardiography, mitochondria, energy metabolism

## Abstract

Myocardial dysfunction is common in septic shock and post-cardiac arrest but manifests differently in pediatric and adult patients. By conventional echocardiographic parameters, biventricular systolic dysfunction is more prevalent in children with septic shock, though strain imaging reveals that myocardial injury may be more common in adults than previously thought. In contrast, diastolic dysfunction in general and post-arrest myocardial systolic dysfunction appear to be more widespread in the adult population. A growing body of evidence suggests that mitochondrial dysfunction mediates myocardial depression in critical illness; alterations in mitochondrial electron transport system function, bioenergetic production, oxidative and nitrosative stress, uncoupling, mitochondrial permeability transition, fusion, fission, biogenesis, and autophagy all may play key pathophysiologic roles. In this review we summarize the epidemiologic and clinical phenotypes of myocardial dysfunction in septic shock and post-cardiac arrest and the multifaceted manifestations of mitochondrial injury in these disease processes. Since neonatal and pediatric-specific data for mitochondrial dysfunction remain sparse, conclusive age-dependent differences are not clear; instead, we highlight what evidence exists and identify gaps in knowledge to guide future research. Finally, since focal ischemic injury (with or without reperfusion) leading to myocardial infarction is predominantly an atherosclerotic disease of the elderly, this review focuses specifically on septic shock and global ischemia-reperfusion injury occurring after resuscitation from cardiac arrest.

## 1. Epidemiology of Sepsis and Cardiac Arrest

Sepsis-3 defined sepsis as life-threatening organ dysfunction caused by a dysregulated host response to infection [1], and this pathophysiologic process represents the major cause of morbidity and mortality in children and adults [2,3,4,5]. The incidence of sepsis is disproportionately increased in elderly adults, and age is an independent predictor of mortality when compared with pediatric or young adult sepsis patients [2,3,6]. While mortality statistics are certainly different between the pediatric and the adult population, children and adult patients often experience cardiovascular dysfunction at a similar rate. In a large, multinational, point prevalence study of severe sepsis, 67% of critically ill children had multi-organ dysfunction at sepsis recognition and 70% had cardiovascular dysfunction in particular [7]. This data is confirmed by other pediatric studies, which suggest the majority of children with septic shock have evidence of myocardial dysfunction [8,9]. Septic myocardial dysfunction is similarly prevalent in adults, with estimates approaching 70% depending on the echocardiographic parameter examined [10,11,12].

Estimates for the incidence of cardiac arrest in the United States are also much higher in adults than in children. Cardiac arrest occurs in hospital in 6000 children each year in the United States [13,14] and out of hospital in another 5000 children [15]. In comparison, out-of-hospital cardiac arrest is estimated to occur in 300,000 adults annually in the United States, while 200,000 adults suffer in-hospital cardiac arrest [16]. There is limited pediatric evidence documenting the incidence of post-arrest myocardial dysfunction, but one study found left ventricular systolic dysfunction in 41% of children after out-of-hospital cardiac arrest [17]. Post-arrest myocardial dysfunction is more common in adults after resuscitation from cardiac arrest, impacting 72–75% of adults after out-of-hospital cardiac arrest in two studies [18,19], though this may relate to the high prevalence of coronary artery disease in this population [20]. Post-cardiac arrest myocardial dysfunction is classically described as a transient phenomenon lasting 24–48 h [21], and recent studies of temporal changes in myocardial function after cardiac arrest support this notion [22,23].

## 2. Clinical Characteristics

Infants and young children with septic shock often present with elevated systemic vascular resistance and low cardiac output in the context of myocardial dysfunction as part of a cold shock phenotype [24,25]. Biventricular dysfunction appears to be common. In a pediatric study in children with fluid- and catecholamine-refractory septic shock, 72% of patients had evidence of left ventricular (LV) dysfunction, while 63% had some degree of right ventricular (RV) dysfunction [8]. Cardiovascular impairment in pediatric septic shock is not limited to systolic dysfunction; estimates for diastolic dysfunction range from 41–48% for the LV and approach 35% for the RV [8,26]. This cardiac dysfunction is also common in neonatal sepsis, as both LV and RV systolic and diastolic dysfunction can occur [27]. Importantly, distinguishing LV and RV impairment and identifying diastolic dysfunction may facilitate more accurate titration of hemodynamic support in children with septic shock [26,28,29]. It must be noted, though, that when speckle tracking echocardiography is employed in children with sepsis and septic shock, impaired ventricular performance is detected, even when the ejection fraction (EF) appears normal, and is associated with worsening of global longitudinal and circumferential strain [30,31]. Whether titrating bedside therapies to improve indices of myocardial strain is of clinical benefit in pediatric septic shock remains an area for future research.

In contrast, adult septic shock is typically characterized by a warm shock phenotype with normal or elevated cardiac output and low systemic vascular resistance [32]. However, EF is a load-dependent measurement and is a potentially misleading metric in states of altered afterload and preload. For example, in a recent study in adult patients with septic shock, Boissier et al. reported that 78% of patients had normal or elevated EF, which manifested with higher cardiac index and lower parameters of afterload, while only 22% of patients presented with low EF [10]. However, when these latter patients were monitored with the application of speckle tracking imaging, a much higher proportion of myocardial dysfunction (>70%) with impairment in global longitudinal strain was observed (Table 1) [10].

As in pediatric septic shock, adult sepsis is characterized by biventricular dysfunction. The prevalence of RV dysfunction ranges from 32–55% and when present is associated with severity of illness and mortality [33,34,35]. Diastolic dysfunction seems to be more prevalent in the adult population, occurring in 66–84% of patients, which may be related to underlying ischemic heart disease, hypertension, and diabetes [12,36]. As previously noted, speckle tracking echocardiography has the potential to reveal myocardial dysfunction not identified by conventional parameters. For example, conventional echocardiography demonstrated LV dysfunction in 33% and RV dysfunction in 32% septic patients, while speckle tracking found LV dysfunction in 69% and RV dysfunction in 72% septic patients [11]. A recent meta-analysis also reported that worse myocardial function as measured by worse global longitudinal strain was associated with higher mortality in patients, while such an association was not valid for LV EF [37]. Taken together, this data suggests that strain analysis by speckle tracking echocardiography may be an important measurement in identifying sepsis-associated myocardial dysfunction in all age groups. Future research should more fully elucidate whether myocardial strain is an appropriate clinical marker to guide treatment in septic shock.

As occurs in septic shock, myocardial dysfunction after resuscitation from cardiac arrest appears to be a global process. A review of echocardiograms of children admitted after out-of-hospital cardiac arrest reported that, in addition to LV systolic dysfunction, 18% of patients had evidence of abnormal RV systolic function, 42% had abnormal septal wall movement, and approximately 65% of patients exhibited diastolic dysfunction of both ventricles [17]. In a study of adult patients with cardiac arrest, RV systolic dysfunction post-arrest was as prevalent as LV dysfunction (59% and 63% patients) and was independently predictive of survival and neurologic outcome [38]. Additional adult data further validates the notion that diastolic dysfunction is common post-arrest and does improve over time in survivors (Table 1) [22]. As in septic shock, identification of LV and RV systolic and diastolic dysfunction after cardiac arrest may be useful in guiding management at the bedside [39]. At present, however, the role of strain echocardiography in delineating myocardial dysfunction and in influencing therapeutic strategies post-cardiac arrest remains unknown and is an important area for future research.

## 3. Biomarkers

Several biomarkers have been examined for their utility in identifying myocardial dysfunction in critical illness and in prognosticating outcome. B-natriuretic peptide (BNP), a hormone secreted by the ventricles in response to wall stretch, and its prohormone have been studied in pediatric and adult septic shock. Domico et al. found that admission BNP was higher in a small cohort of children with septic shock than in controls and was higher in cold shock than in warm shock; BNP was inversely correlated with fractional shortening and directly correlated with severity of illness [40]. This is further corroborated by pediatric data showing correlations of BNP with LV systolic dysfunction and with severity of illness in septic shock [8]. Thus, BNP may aid in the identification of children with sepsis-associated myocardial dysfunction, though temporal relationships with treatment and clinical improvement remain undefined.

The utility of BNP in adult sepsis, on the other hand, appears to vary in relation to the clinical course of illness. When examined in the Emergency Department in patients with suspected sepsis, elevated BNP was associated with mortality but had limited diagnostic utility in predicting progression to shock [41]. When examined in adults with confirmed sepsis during hospitalization, BNP levels correlated with tissue hypoxia, higher vasoactive use, lower EF, and mortality [42,43]. Conversely, improvement in BNP portends the return of myocardial function and survival [42,44], suggesting a potential role for BNP in monitoring patient response to the treatment of septic shock.

The role of BNP after cardiac arrest is less clear; BNP does not seem to be associated with the presence of LV systolic dysfunction in children after cardiac arrest [17], though elevated admission BNP is associated with unfavorable outcomes in adults after out-of-hospital cardiac arrest [45]. Currently, BNP may be more helpful in the identification of myocardial dysfunction in children and adults with septic shock, rather than after resuscitation from cardiac arrest.

Cardiac troponin is essential for muscle contraction and ventricular function, binding intracellular calcium to regulate the interaction between myosin and actin. In particular, cardiac troponin subunits T and I have also been extensively studied as biomarkers of myocardial dysfunction in critical illness. Cardiac troponin-T correlates with LV and RV systolic dysfunction and with mortality in late-onset neonatal sepsis [27]. Troponin-T has also been associated with LV diastolic dysfunction, RV dilation, and LV global longitudinal strain in adult septic shock [46,47]. An estimated two-thirds of adults with septic shock who have elevated high-sensitivity troponin-I have myocardial dysfunction, and troponin-I correlates with mortality in these patients [44,48]. The utility of troponin measurement in pediatric septic shock is less clear, however; although elevated troponin-T is common in children with septic shock who have myocardial dysfunction, this elevation does not appear to have a clear association with diastolic dysfunction on multivariable analysis [26]. Future research will have to determine whether troponin measurement has a role in the identification of myocardial dysfunction and in prognostication in children with septic shock. Unlike BNP, troponin is associated with LV systolic dysfunction in children resuscitated from cardiac arrest [17]. In adults with out-of-hospital cardiac arrest, both troponin-T and -I are elevated and are not necessarily indicative of antecedent coronary occlusion (Table 1) [19,22]. Clinicians should thus be cautious in interpreting cardiac troponin levels in older patients in whom myocardial infarction may have precipitated cardiac arrest.

A few other biomarkers have been evaluated in adults with severe sepsis and septic shock, though little evidence exists at present to support their use. Secretoneurin and chromogranin A, both produced in myocardial and neuroendocrine tissue, have been correlated with mortality in adult septic shock [49,50]. Neutrophil gelatinase-associated lipocalin, often associated with acute kidney injury, is also associated with low EF, vasoactive support, and mortality in adults with severe sepsis or septic shock [51]. It remains to be seen if monitoring of any of these biomarkers offers any marginal benefit above that of BNP and cardiac troponins in septic shock and after resuscitation from cardiac arrest.

## 4. Energy Failure and Mitochondrial Dysfunction

**Figure 1:**
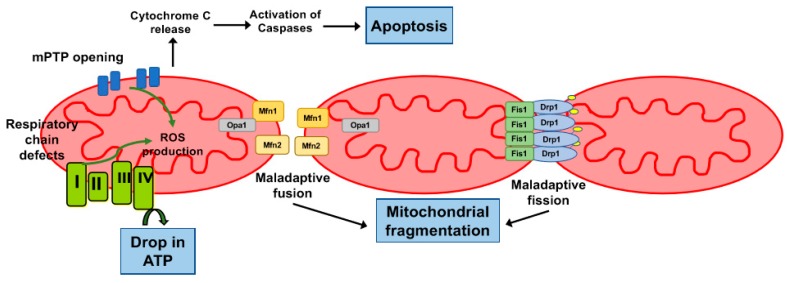
Mitochondrial dysfunction in cardiomyocytes in sepsis and cardiac arrest. Defects in activity of complexes I, II, and IV lead to a reduction of adenosine triphosphate (ATP) and an increase in reactive oxygen species (ROS). Other downstream consequences of mitochondrial permeability transition pore (mPTP) opening are cytochrome C release, increased caspase activity, and apoptosis. Opening of the mPTP contributes to energy failure. Defects in mitochondrial dynamics lead to mitochondrial fragmentation. Defects are related to increased expression of dynamin-related protein 1 (Drp1). Drp1 interacts with mitochondrial adaptor fission 1 (Fis1) on the outer mitochondrial membrane to facilitate mitochondrial fission. Inhibition of expression of mitofusin 1 (Mfn1) and mitofusin 2 (Mfn2), necessary for outer mitochondrial membrane fusion, as well as optic atrophy 1 (Opa1), which localizes to the inner mitochondrial membrane, leads to reduction of mitochondrial fusion.

Mitochondrial dysfunction has been proposed as an important cause of sepsis-related organ failure, and mitochondrial ultrastructural damage and diminished adenosine triphosphate (ATP) production have been demonstrated in cardiomyocytes during sepsis in experimental animals and humans (Figure 1) [52,53,54,55,56]. One of the major pathophysiological events occurring in sepsis is impairment of mitochondrial respiratory chain complex activity and bioenergetic production. For example, polymicrobial sepsis, induced by cecal ligation and puncture (CLP), in young adult mice results in diminished myocardial complex IV (CIV, cytochrome c oxidase) activity for up to 48 h, accompanied by decreased expression of CIV subunits [54,55]. Restoration of CIV activity with exogenous administration of cytochrome c improves survival after CLP, suggesting a potential causative role for mitochondrial dysfunction in mortality in sepsis [55]. Sepsis in young adult mice also results in decreased complex I (CI)- and complex II (CII)-driven respiration and reduction in maximum oxidative phosphorylation (OXPHOS) [57]. Since antioxidant administration with melatonin rescued CI, CII, and maximum OXPHOS in this model, oxidative and nitrosative damage may play a role in mediating mitochondrial dysfunction [57]. Supporting this notion, cardiac-specific overexpression of thioredoxin, a thiol-based antioxidant, rescues myocardial CI dysfunction [58]. Porcine models of young adult sepsis have also revealed CII and CIV dysfunction 24 h after onset of sepsis, correlating with impairments in myocardial stroke volume [59]. The heterogeneity in the sites of electron transport system (ETS) inhibition may reflect differences in animal selection, models for sepsis induction, and timing of measurements, but the aggregate evidence suggests that impairment in mitochondrial respiration occurs commonly in the septic myocardium.

The downstream effects of diminished mitochondrial respiration are alterations in myocardial bioenergetics. Endotoxin administration in adult rats leads to decreased myocardial ATP production and reduction in high-energy phosphate reserves in the form of creatine phosphate at early and late time points, in addition to myocardial dysfunction and inhibition of CI, CII, and CIV [60,61]. These changes are also accompanied by increased production of reactive oxygen species (ROS) and nitric oxide, further suggesting a possible link between CI dysfunction and nitrosative and oxidative injury [61]. Rats with polymicrobial sepsis exhibit a similar decline in myocardial ATP, creatinine phosphate, and glycogen stores [62], suggesting this phenotype is not unique to endotoxin models of sepsis. Since cardiac muscle contraction is an ATP-dependent process, these findings further add to the pathophysiologic link between mitochondrial dysfunction and sepsis-induced myocardial dysfunction.

However, it must be noted that all of these experimental studies have used models in young adult animals, and very few studies have directly compared multiple age groups. In a recent study from our laboratory, we have found that sepsis-induced myocardial dysfunction manifests with age-dependent characteristics, which are associated with a distinct metabolic dysregulation [63]. For example, middle-age mice (9–12 months old) exhibited more severe cardiac injury and worse mitochondrial derangement as compared to a younger group of mice (2–3 months old) despite equivalent troponin-I and -T levels after sepsis. Echocardiography revealed that young mice exhibited left ventricular systolic dysfunction, whereas middle-age mice exhibited reduction in stroke volume without apparent changes in load-dependent indices of cardiac function [63]. Other studies in six-week-old and twenty-month-old mice have demonstrated distinct age-dependent alterations in gene expression pathways related to amino acid and fatty acid metabolism, which can impact cellular bioenergetics after sepsis [64]. It is also known that reduced activity of the mitochondrial ETS and reduced cytochrome P450 expression in the myocardium also occur as part of normal aging [65], which may exacerbate the degree of mitochondrial dysfunction in septic shock. These unique transcriptional changes are also responsible for excessive ROS production by the mitochondria, thus becoming an important cause of enhanced susceptibility to myocardial dysfunction in adult and elderly patients [65,66].

Mitochondrial injury has also been clearly demonstrated in the myocardium after cardiac arrest. In a model of pediatric cardiac arrest using four-week-old piglets, CI dysfunction persisted in both the LV and RV in survivors at 24 h, and the degree of CI dysfunction depended on the coronary perfusion pressure, a driver of coronary blood flow, during cardiopulmonary resuscitation [67], linking the extent of mitochondrial dysfunction with the severity of the ischemic injury to the myocardium. Similarly, young adult murine models of cardiac arrest have revealed early CI and CIV dysfunction in the context of ROS and reactive nitrogen species (RNS) generation [68,69]. Interestingly, in contrast to sepsis-induced myocardial dysfunction, CII dysfunction is not commonly reported after resuscitation from cardiac arrest; at present, it is unclear if and why sepsis may be more likely to induce CII dysfunction in the myocardium, in addition to CI and CIV dysfunction. As in septic shock, however, cardiac arrest also leads to diminished myocardial adenosine diphosphate (ADP) and ATP with increased intramyocellular lipid droplets and glycogen deposits suggestive of impaired substrate utilization [70]. While ETS dysfunction is evident in both juvenile and adult models of cardiac arrest, no studies as yet have directly compared mitochondrial function in survivors of cardiac arrest of different ages, and this remains an important area for future research.

As noted in the above discussion, oxidative and nitrosative damage may play a key pathophysiologic role in mitochondrial respiratory chain dysfunction in the heart in both septic shock and after cardiac arrest. ROS are generated at multiple sites in the mitochondria, including through electron leak at CI, CII, and complex III (CIII) to oxygen, creating superoxide radicals and hydrogen peroxide [71]. Impairment in respiratory chain function may encourage additional electron leak and ROS generation upstream in the ETS. Furthermore, reverse electron transport has been suggested as an important factor in ROS generation, with ischemic accumulation of succinate driving reverse electron transport from CII to CI at reperfusion [72]. This may be a particularly important mechanism in the myocardium after return of spontaneous circulation (ROSC) is achieved post-cardiac arrest, but whether this contributes to the pathophysiology of the septic heart remains poorly defined. Excessive ROS generation causes oxidative stress and impairs mitochondrial function, in part through oxidation of cardiolipin, an inner mitochondrial membrane phospholipid. This disrupts ETS complexes and may lead to the opening of mitochondrial permeability transition pores (discussed further below) [73]. Excess nitric oxide production also occurs in the myocardium in septic shock [74] and cardiac arrest [68], generating RNS such as peroxynitrite through interaction with superoxide radicals. This nitrosative stress also impairs mitochondrial ETS function, as peroxynitrite is a potent irreversible inhibitor of CI [75]. However, studies examining the influence of age on myocardial ROS and RNS generation in septic shock and post-cardiac arrest are lacking.

Finally, it should also be noted that mitochondrial uncoupling impacts ROS generation and may thereby influence oxidative damage in the septic myocardium and post-cardiac arrest. Mitochondrial ROS generation correlates with the proton-motive gradient; as respiration slows at higher proton-motive force, electron leak increases at CI, CII, and CIII [76]. Basal proton leak across the inner mitochondrial membrane dissipates this proton-motive force, and proton leak can be augmented by a family of inducible uncoupling proteins (UCP) [76,77]. Increased uncoupling, then, has the potential to limit ROS generation by the mitochondria, at the cost of diminishing ATP production. Whether UCP play a protective or pathophysiologic role in the heart post-cardiac arrest is largely unexplored, though there is emerging evidence that uncoupling protein 2 (UCP2) may be protective in the septic myocardium [78,79]. Once again, though, the extent and function of mitochondrial uncoupling in neonatal and pediatric myocardium in sepsis and cardiac arrest remains an area for future research.

## 5. Role of the Mitochondrial Permeability Transition Pore

The mitochondrial permeability transition pore (mPTP) is a heterogenous complex in the inner mitochondrial membrane whose composition has not yet been fully elucidated [80] but which is thought to contribute to myocardial dysfunction in septic shock and as part of ischemia-reperfusion injury occurring with resuscitation from cardiac arrest. While transient opening of the mPTP has been linked to physiologic ROS signaling and mitochondrial calcium efflux, prolonged opening of the mPTP leads to inner mitochondrial membrane depolarization, abolishing the proton-motive gradient necessary for ATP synthesis and thereby causing bioenergetic depletion of the cardiomyocyte [81,82]. Prolonged opening is also accompanied by mitochondrial swelling and rupture and release of pro-apoptotic factors such as cytochrome c, ultimately leading to apoptotic or necrotic cell death if mPTP opening extends to a significant portion of the cellular mitochondrial network [83,84] (Figure 1).

Notably, however, cardiomyocyte cell death is rarely observed in post mortem hearts from septic adults, despite the magnitude of organ dysfunction [56]. This suggests that widespread irreversible mPTP opening does not routinely occur in sepsis-associated myocardial dysfunction. Nevertheless, there is evidence implicating either 1) potentially reversible alterations in the mPTP or 2) irreversible mPTP opening in limited numbers of mitochondria in the pathogenesis of myocardial dysfunction in animal models of sepsis. Endotoxin-treated cardiomyocytes exhibit mPTP opening with mitochondrial membrane depolarization and ROS generation, which is ameliorated by treatment with cyclosporine A, an mPTP inhibitor [85]. Furthermore, inhibition of mPTP opening in both endotoxin and CLP models of adult sepsis improves OXPHOS, myocardial function, and survival [86,87,88]. These findings point to pathophysiologic changes in mPTP opening in the septic heart that fall short of catastrophic bioenergetic failure and initiation of cell death in most circumstances. It must be noted that all of these studies included only adult cardiomyocytes or adult animals; thus, the effects of mPTP opening on the juvenile myocardium in sepsis remains largely unexplored.

mPTP opening is similarly thought to be involved in myocardial dysfunction occurring after resuscitation from cardiac arrest. In fact, ROSC is associated with mPTP opening within minutes of reperfusion after the ischemic period. The mPTP is thought to remain closed during ischemia due to profound intracellular acidosis; with normalization of pH after reperfusion, the mPTP opens in response to intracellular calcium overload, ATP depletion, and oxidative stress [89,90]. This in turn arrests mitochondrial respiration by abrogating the proton gradient across the inner mitochondrial membrane, thereby stopping further ATP synthesis. Even so, as in septic shock, cardiomyocyte death is rare in survivors of cardiac arrest [91], arguing against widespread irreversible changes in the mPTP, which would be expected to activate cell death pathways. Lack of significant myocardial cell death in septic shock and post-cardiac arrest is also consistent with the known clinical course in survivors with recovery characterized by normalization of myocardial function. However, the degree of ischemia time prior to ROSC does seem to impact the degree of mPTP opening, with prolonged ischemic periods causing more changes in the mPTP [92]. Finally, the etiology of cardiac arrest may further impact the extent of mPTP opening, as asphyxial cardiac arrest appears to be associated with more extensive mPTP opening than does ventricular fibrillation cardiac arrest [69].

As in the septic myocardium, this transient or limited mPTP opening appears to play a pathophysiologic role in myocardial dysfunction post-cardiac arrest. After 15 min of untreated cardiac arrest in rabbits, administration of mPTP inhibitors improved myocardial function, lowered troponin leak, and improved maximum OXPHOS [89]. mPTP inhibition with cyclosporine has also been noted to improve both myocardial function and survival in adult rats, though administration must occur prior to ROSC to be efficacious, since reperfusion initiates mPTP opening [93]. While most studies have been performed in adult models of cardiac arrest, mPTP opening has been identified in the neonatal myocardium as well. It must be noted, however, that studies of neonatal cardiac arrest have focused on cardioplegic arrest in relation to cardiac surgery with cardiopulmonary bypass. Unlike in post-cardiac arrest myocardial dysfunction, apoptosis in the context of mPTP opening is much more common in the neonatal myocardium after cardioplegic arrest [94]. Inhibition of mPTP opening in neonatal cardioplegic arrest in rabbits with cyclosporine A improves myocardial function and CI respiration and reduces ROS production [94,95]. In a direct comparison of neonatal (6–8 days) and young adult (6–8 weeks) lambs who underwent cardioplegic arrest for cardiopulmonary bypass, neonatal myocardium exhibited more evidence of mPTP opening, characterized by increased cytochrome c release, caspase activity, and apoptosis [96]. At present, it is unclear whether this increased susceptibility to mPTP opening and apoptosis in the neonatal myocardium extends to cardiac arrest in general or if this remains limited to cardioplegic arrest for cardiac surgery. Further research is required to better delineate mPTP opening in the neonatal and pediatric myocardium in both septic shock and post-cardiac arrest. Cyclosporine A has recently been investigated as a therapeutic agent in adults post-cardiac arrest [97], but the multifaceted effects of calcineurin inhibitors such as cyclosporine complicate translation to human clinical trials.

As mPTP opening is triggered in the context of mitochondrial calcium overload, dysregulation in intracellular calcium can have potentially deleterious effects on the myocardium. At physiologic levels, calcium accelerates oxidative phosphorylation through stimulation of both Krebs cycle enzymes as well as complex V (ATP synthase) of the ETS [98]. Coupling of energy demand for calcium-dependent muscle contraction with energy production occurs in this way through close approximation of cardiac mitochondria with the sarcoplasmic reticulum, thought to be mediated by mitofusin-2 (discussed further below) [99]. An extensive discussion of intracellular calcium homeostasis is outside the scope of this review and is covered well elsewhere [99,100], especially since few studies have examined mitochondrial calcium in the heart in septic shock and post-cardiac arrest and since neonatal and pediatric data are absent.

## 6. Mitochondrial Dynamics in the Myocardium

Emerging evidence suggests that disruption of normal mitochondrial fission and fusion, collectively termed mitochondrial dynamics and important for homeostasis of the intracellular mitochondrial network, contribute to organ failure in sepsis [101,102]. Mitochondrial fission mediates both mitochondrial replication and the sequestration of damaged parts of mitochondria by recruitment of dynamin-related protein 1 (Drp1) to the outer mitochondrial membrane [103]. Conversely, mitochondrial fusion is crucial for maintenance of the mitochondrial membrane potential and may rescue the function of damaged mitochondria. In addition to its role in tethering cardiac mitochondria to the sarcoplasmic reticulum, mitofusin 2 (Mfn2) and its counterpart mitofusin-1 (Mfn1) facilitate outer mitochondrial membrane fusion, while optic atrophy 1 (Opa1) protein is involved in fusion of the inner mitochondrial membrane (Figure 1) [103].

There are as yet no neonatal or pediatric-specific studies examining mitochondrial dynamics in the myocardium during sepsis, but data from adult models suggest the involvement of multiple signaling pathways and highlight the importance of the relative balance of fission and fusion [58]. Endotoxin administration in adult male mice results in increased Drp1 activation by phosphorylation (Ser616) in the myocardium, leading to mitochondrial fragmentation and decreases in CI, CII, and CIV-driven respiration and in left ventricular function [104]. Inhibition of the RhoA/ROCK (Ras-homologous A/RhoA-associated coiled-coil-containing protein kinases) pathway decreased Drp1 phosphorylation, normalizing mitochondrial length and improving respiration and myocardial function [104], while inhibition of the JNK-LATS2 (c-Jun N-terminal kinase/large tumor suppressor kinase 2) pathway reduced Drp1 expression and restored Opa1 and Mfn2 expression with improvement in myocardial function, ATP content, and mitochondrial size [105]. Similarly, Mst1 (Mammalian sterile 20-like kinase 1) activation seems to be involved in upregulation of Drp1 and suppression of Mfn1, Mfn2, and Opa1, leading to mitochondrial fragmentation in the myocardium after endotoxin exposure [106]. Finally, the interaction of Drp1 with mitochondrial adaptor fission 1 (Fis1) seems necessary for maladaptive fission in endotoxin models of sepsis, as inhibition of this interaction reduces mitochondrial fragmentation, myocardial dysfunction, and mortality [107]. The myriad pathways involved underscore the complexity of control over mitochondrial fission and fusion, but the aggregate evidence indicates a potentially pathologic role for mitochondrial fission in the adult septic heart.

Pathologic mitochondrial fission may also contribute to myocardial dysfunction after resuscitation from cardiac arrest in both neonates and adults. In neonatal murine cardiomyocytes, Drp1 inhibition preserved mitochondrial morphology and respiration after global ischemia-reperfusion injury; similarly, Drp1 inhibition prior to or following global ischemia-reperfusion in adult rat hearts reduced ROS generation and improved diastolic function [108]. Furthermore, Drp1 inhibition during cardiopulmonary resuscitation in a murine adult model of cardiac arrest normalized mitochondrial morphology in the myocardium, reduced time to ROSC, and improved post-resuscitation myocardial function and survival [109]. These promising data underscore the need for additional research to better delineate the role of mitochondrial dynamics in the myocardium both during sepsis and after resuscitation from cardiac arrest in all age groups and to outline the therapeutic potential of pharmacologic modulation of these processes.

In addition to mitochondrial fission and fusion, mitochondrial turnover is also mediated by autophagy and mitochondrial biogenesis. Autophagy mediates the removal of damaged organelles through lysosomal degradation [110], and mitophagy is the term applied to the targeted autophagy of damaged mitochondria before cell death pathways can be triggered by mitochondrial permeability transition [111]. In contrast, mitochondrial biogenesis, largely regulated by peroxisome proliferator-activated receptor (PPAR)-γ coactivator-1 (PGC-1) α and β, refers to the generation of new mitochondria [112]. An extensive review of mitophagy and mitochondrial biogenesis is outside the scope of this review, but mitophagy may be crucial in cardiac recovery after resolution of sepsis [83], and biogenesis may be modulated in the septic myocardium [113]. Interestingly, these processes seem to be more robust in the neonate, as the resistance of neonatal cardiomyocytes to apoptosis has been attributed to both increased mitochondrial biogenesis and mitophagy [114]. Whether mitochondrial biogenesis and mitophagy play a larger role in neonatal and pediatric hearts in sepsis merits further exploration. Mitochondrial biogenesis and mitophagy are also being increasingly explored in post-cardiac arrest myocardial dysfunction [115,116], but studies in younger patients are lacking.

## 7. Conclusions

Myocardial dysfunction consequent to sepsis or cardiac arrest is associated with profound changes in cardiac metabolism. Experimental data from animal models have revealed that mitochondrial dysfunction contributes to loss of cardiac metabolic flexibility and to contractile impairment. At present, however, most of the literature reports data from experimental models using young adult animals, which correspond in human age to adolescents or young adults and, therefore, poorly correlate with older adult or elderly sepsis. There is also a paucity of pre-clinical studies examining myocardial dysfunction and mitochondrial alterations in neonatal and pediatric age groups. Future research into myocardial dysfunction in sepsis and after cardiac arrest should employ models of multiple ages across the lifespan from early development to senescence to both further define age-specific differences in cardiac injury and to account for potential age-dependent responses to therapeutic interventions.

## Figures and Tables

**Table 1:** Characteristics of myocardial dysfunction in septic shock and post-cardiac arrest.

**Septic Shock**		
**Myocardial Dysfunction**	**Pediatrics**	**Adult**
LV systolic	3/4 of patients	Low EF in 1/4 to 1/3 *
RV systolic	2/3 of patients	32–55% of patients *
Diastolic	41–48% for LV ~35% for RV	66–84% of patients
**Biomarkers**		
B-natriuretic peptide	↑ with LV dysfunction	↑ with ↓ EF
Cardiac troponins	↑ with LV and RV dysfunction in neonates	↑ with LV dysfunction
**Post-Cardiac Arrest**		
**Myocardial Dysfunction**	**Pediatrics**	**Adult**
LV systolic	41% (single study)	>50%
RV systolic	18% (single study)	>50%
Diastolic	~2/3 for LV and RV	Common
**Biomarkers**		
B-natriuretic peptide	No clear association	No clear association
Cardiac troponins	↑ with LV dysfunction	Not necessarily indicative of coronary occlusion

Definitions of abbreviations: EF = ejection fraction; LV = left ventricle; RV = right ventricle. * Strain imaging suggests systolic dysfunction may be much more prevalent (>70%).
